# Navigating financial coverage of healthcare to undocumented migrants at two hospitals in Oslo: a qualitative study

**DOI:** 10.1177/14034948251318512

**Published:** 2025-02-16

**Authors:** Kristine Fjelltorp-Veland, Heidi E. Fjeld, Frode Eick

**Affiliations:** 1 Department of Community Medicine and Global Health, Institute of Health and Society, University of Oslo, Norway; 2 Lovisenberg Diaconal University College, Oslo, Norway

**Keywords:** Insurance coverage, undocumented migrants, guidelines, othering, access to healthcare

## Abstract

**Aims:**

This study aimed to investigate how hospitals navigate financial coverage of healthcare for undocumented migrants, given the present laws and regulations in Norway.

**Methods:**

This qualitative study used an explorative approach. We collected and studied hospital guidelines for registering and invoicing foreign patients, and interviewed hospital staff from two university hospitals, and undocumented migrants at one non-governmental clinic in Oslo. The first author collected 14 documents and conducted 14 semi-structured, in-depth interviews. The project team used a thematic-analysis-inspired approach to identify patterns of shared meaning in the guidelines and interviews.

**Results:**

We found that the hospital guidelines did not account for undocumented migrants. The staff had to navigate between the guidelines and practical implications of undocumented migrant patients not having a resident permit and thus lacking a Norwegian identity number, bank card, or address. We found discrepancies between different staff’s expected roles in the registration of patients and in the assessment of patients’ ability to pay. The guidelines presumed an active patient and required documentation, which undocumented migrants had difficulty meeting.

**Conclusions:**

The underlying assumption of patients being documented in routines led to a process of othering of undocumented patients and thereby reproducing their marginalised position in the health system, hence depriving them of the right to ‘health care that is absolutely necessary and cannot wait’. We recommend that hospitals increase staff’s knowledge and capacity to ensure undocumented migrants’ right to healthcare.

## Background

Nordic countries have, in varying ways, restricted healthcare to undocumented migrants, that is, persons without the appropriate documentation to stay in a country [1]. Restricted or partial rights can be vague and unclear for both patients and health professionals; however, it is essential to ensure that these rights are respected and granted in practice [2, 3]. Since 2011, undocumented migrants in Norway have had the right to emergency care and ‘health care that is totally necessary and cannot be deferred’ including extended rights related to communicable diseases, pregnancy and children [4].

Financial constraints have proven to be both a barrier as well as a heavy burden for undocumented migrants seeking healthcare [4, 5]. Whereas Sweden has granted financial coverage of the healthcare that undocumented migrants are entitled to, this is not the case in Denmark and Norway [6, 7]. Undocumented migrants are left out of the national insurance scheme and should, by default, pay out of pocket for all healthcare provided, except that provided to migrant children [8]. However, asking for prepayment for emergency and ‘health care that is totally necessary and cannot be deferred’ is not allowed, and the hospitals should cover the costs of the provided specialised care if the patient cannot pay [8]. Hospitals’ negotiation of payment for healthcare to undocumented migrants in a high-income country such as Norway is crucial for their access to essential care.

According to earlier reports by social and health professionals in Nordic countries, the hospital staff who have to navigate between patients’ medical needs, the regulations and internal guidelines can face several challenges [9-12]. As migration control has changed from the checkpoints at the national border to the responsibility of international parties (such as the EU and Schengen), private parties (such as airlines and employers), and local authorities and welfare services (such as healthcare), health professionals may indirectly become entangled in migration control [13]. Health professionals may also develop an alternative practice and ethics when encountering undocumented migrants [14]. Hospital staff in Norway have raised their concerns about unstandardised and unclear routines for payment in the hospital system for undocumented migrants. Hence, it is essential to identify the main factors contributing to the possible arbitrary treatment and lack of rightful access.

We will use the theory of othering [15] as a theoretical framework to discuss the themes in the current study. Othering as a concept involves drawing a line ‘between “us” and “them” – between the more and the less powerful – and through which social distance is established and maintained’ [16]. Several micro-practices of border controls may present as a barrier to healthcare for undocumented migrants, such as the fact that basic technology used within the healthcare system requires a Norwegian identity number [17]. Undocumented migrants have different rights to healthcare, lack insurance and a Norwegian identity number, which then requires ad hoc navigation from both the patient and the healthcare professionals. It is therefore necessary to gain more insight into how hospital staff navigate between healthcare needs, rights to healthcare and payment for services.

## Aims

This study aimed to investigate how hospital staff navigate the issue of payment of healthcare for undocumented migrants, given the present laws and regulations in Norway.

## Methods

### Design

As the study was investigating a problem with little to no past data to rely on, we used an explorative approach.

### Setting

According to Statistics Norway, the capital of Norway, Oslo, has 699,827 inhabitants per 01.01.2022 [18]. It is the municipality with the highest proportion of immigrants (25.4%) in Norway [19]. The welfare system in Norway is founded upon citizens’ membership in the national insurance scheme verified through a Norwegian identity number and a registered address in the National Population Register. Hospitals are mainly public, governed and financed through regional health trusts [20]. This study was initiated by a collaboration of Oslo University Hospital (OUS), Akershus University Hospital (Ahus), the two main hospitals in the region of Oslo, and the non-governmental (NGO) clinic serving undocumented migrants in Oslo.

### Participants

Nine hospital staff and five undocumented migrants participated in the study. Out of the nine, five were administrative staff (health secretaries, staff from the international office (only at OUS), and the accounting department), and four were nurses. Hospital staff were recruited through hospital staff facilitating the study, the NGO clinic and personal nursing networks. The NGO clinic recruited five undocumented migrants who had lived between 2 and 20 years in Norway, had been admitted to a hospital and had settled a payment issue with them.

### Data collection

The first author collected data from documents and interviews between November 2021 and February 2022. Hospitals shared their guidelines and other relevant documents on payment from foreign nationals. Because both hospitals had been working on improving their system for handling undocumented migrants, the guidelines collected were newly updated. The NGO clinic also shared its guidelines and documents concerning communication with hospitals relating to undocumented migrants’ invoices.

The first author conducted semi-structured, in-depth interviews with open-ended questions to allow for elaborations (Supplementary File). Two of the hospital staff were interviewed in person, and seven were interviewed online due to the COVID-19 restrictions. The undocumented migrants were interviewed in English or Norwegian in person at the NGO clinic. All interviews were recorded through an application (Nettskjema) directly connected to the Services for Sensitive Data at the University of Oslo. Interviews were anonymised and transcribed on a rolling basis throughout the data collection period.

### Analyses

The analysis was inspired by what Braun and Clarke now conceptualise as reflexive thematic analysis and followed the standardised phases of their initial paper about thematic analysis [21, 22]. Due to the explorative methodology of the project, we developed the codes and themes inductively, aiming for new insights into how hospital staff navigate the issue of payment of healthcare for undocumented migrants. The first author wrote reflective memos throughout the project, which were shared and discussed with the co-authors. We concurrently analysed the documents and transcribed the interviews. In the analysis process we developed an overview of the financial process in the hospitals, from registering to invoicing and handling the invoices, by first closely studying the documents provided by OUS and then proceeding to the documents provided from Ahus. The data material was coded using NVivo. The codes that appeared to be most meaningful and/or had the most entries were categorised into the main themes describing the financial process and encounters between staff and undocumented migrants in the hospitals, and that explained the core challenges within these.

It is worth noting that two of the authors are health professionals, and two had previously been involved in research about undocumented migrants. We therefore acknowledge our positionality of advocating for the right to healthcare of undocumented migrants. Our previous work within hospitals and our extensive experience from providing healthcare to, and researching about, undocumented migrants in Norway enabled well-informed and nuanced readings of the results and the generating of main themes through a process of thematic-analysis-inspired interpretations and meaning-making.

### Ethics

The study was conducted in accordance with the Declaration of Helsinki. The project received partial approval on the 18 October 2021 and final approval from the Norwegian Centre for Research Data on the 11 January 2022 (project number: 360058). A combined information and consent sheet were sent to the hospital staff before the interview. Undocumented migrants received the information and consent sheet in English or Norwegian before the interview. Hospital staff gave written consent, and undocumented migrants gave oral consent to participate in the study.

## Results

We generated four main themes from the analysis: (1) identity, documentation requirements and address as barriers, (2) fluctuating responsibility for assessment of solvency, (3) rights versus payment, and (4) the presumption of an active patient. The guidelines did not account for undocumented migrants. Hospital staff were uncertain about their role in the registration and how to assess whether or not undocumented migrant patients should pay for the healthcare received. The staff had to navigate between the guidelines and the practical issues of registration, such as the patient not having a Norwegian identity number, bank card or address. The guidelines presumed an active patient and required documentation, which undocumented migrants had difficulty meeting.

### Identity, documentation requirements and address as barriers

**Figure 1.**
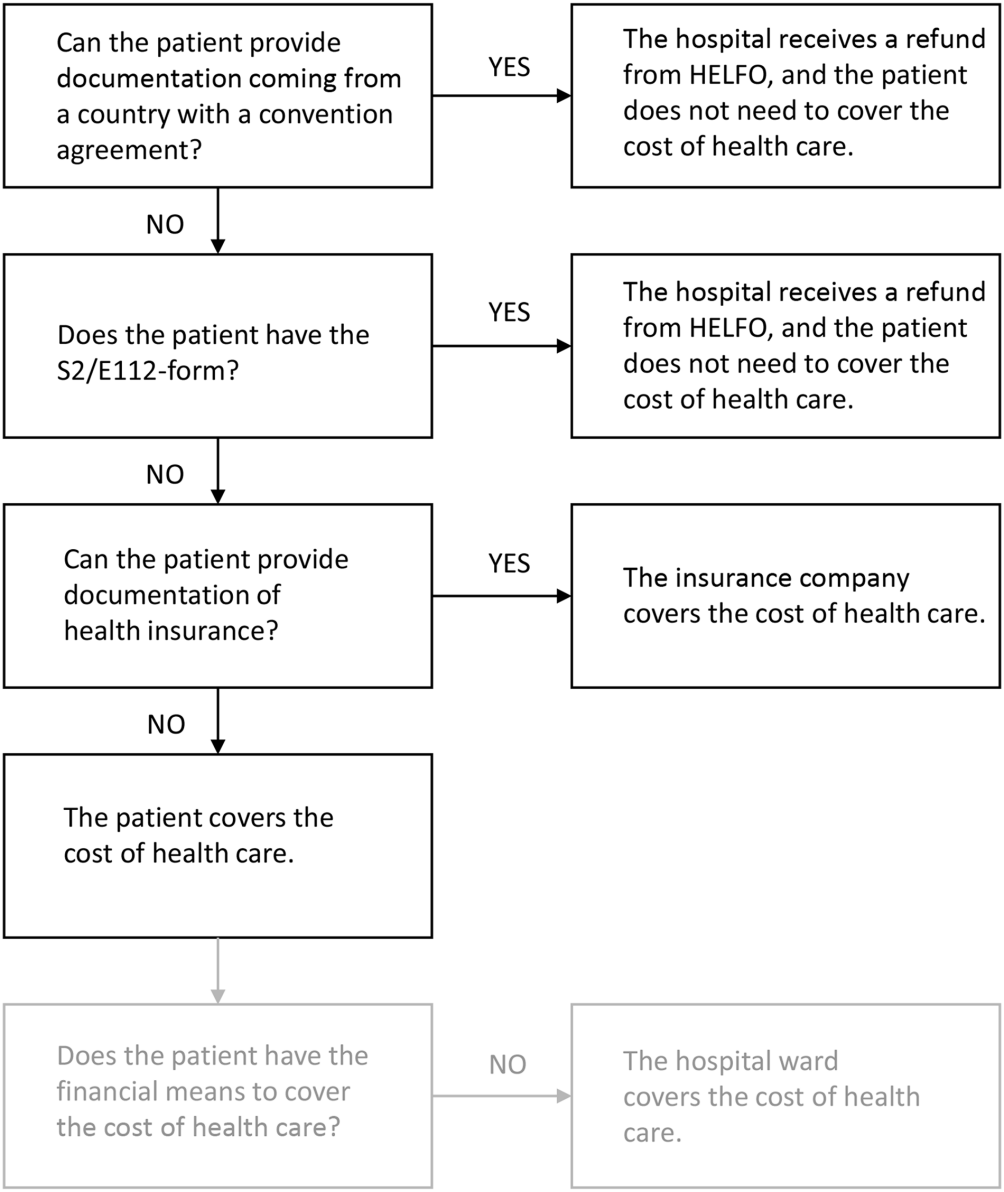
Payment guidelines for foreign patients at two Norwegian hospitals.

It was recurrent through the guidelines that foreign patients had to show documents of membership in national insurance schemes in countries that have convention agreements with Norway, private health insurance or other sources of coverage. The hospital staff reported that most foreign patients could document being members of schemes through which they were entitled to have the treatment covered (Figure 1). Except for one administrative notice, the guidelines did not address the possibility of patients lacking these required documents and hence gave no guidance about required action in those cases. However, we found that it was possible to register the patient in the electronic patient record system without documentation of identification or address.

### Fluctuating responsibility for assessment of solvency

**Figure 2.**
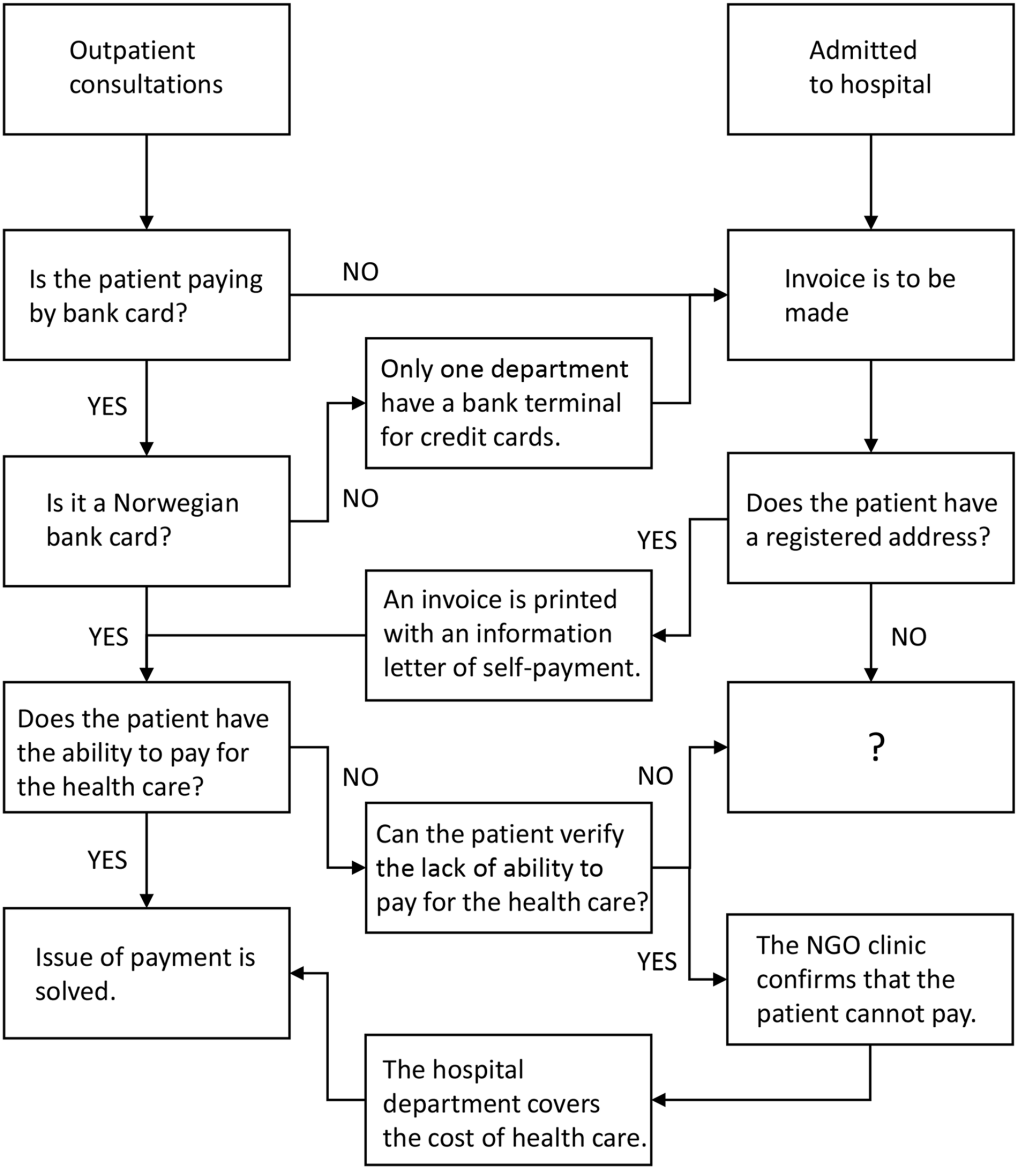
Process of invoicing self-paying patients.

The actors involved in the registration and invoicing of patients included the international office, the accounting department, as well as healthcare professionals and health secretaries at the ward where the patient received treatment. The different actors viewed their roles in registration and invoicing differently. The hospital’s guidelines stated, ‘If the patient cannot pay, the hospital has to cover the expenses’. However, none of the guidelines indicated how the patient’s ability to pay should be assessed, nor whose responsibility it was to do the assessment (Figure 2). Hospital staff frequently repeated in the interviews that they found that the patients claims that they were unable to pay were insufficient for a decision. They found the distinction between not wanting to pay and being unable to pay to be unclear. An employee at the international office expressed their role:

But as it is now, it is the hospital that must cover it [the treatment for patients without the ability to pay], and that is very difficult, especially for the wards, to kind of stop the obligation to pay already there and then, when the patient says, ‘I cannot pay’. Because we do not have a scheme covering them, we must be as strict because we [with strong emphasis] must follow the regulations. Therefore, in my position, economics take precedence.

An employee at the accounting department expressed that the staff at the ward should have an opinion about whether a patient can pay for the treatment or not and stated: ‘We are in accounting; we only execute this [the invoicing]’. The health secretaries received questions about payment from the accounting department and from management and experienced that healthcare professionals were unaware of whether a patient had the necessary documentation or not. Health secretaries reported that they printed an invoice in the cases where they were unsure about the patient’s insurance status, and thought about this as not taking a stand on the question of the patient’s ability to pay.

Healthcare professionals also expressed that assessing a patient’s ability to pay was outside their area of competence, and they did not pay much attention to the question because, ‘it is someone else who takes care of it’. As health professionals they were trained to give the best treatment possible, no matter the patient’s situation. On the other side of these encounters, an undocumented migrant experienced it like this:

. . . there came a person called social worker, someone in charge of those payments. We talk about the bills and the payment. She understands my problem that I don’t have the . . . I’m waiting for the decision of UDI (immigration authorities). She will try to see how they can deal with the bills that are in the future, that they will not come. But the bills that were there already, they cannot do anything. [. . .] But to me, the main problem, I think about those bills that were in debt collection already. They cause problems. They tell me it is a big problem.

Most of the administrative staff at the hospitals held the view that the NGO clinic should assess the ability to pay because they knew the patient best. We found that if the hospitals received an inquiry from the NGO clinic confirming the patient’s lack of ability to pay, they would credit the invoice without further questions.

### Rights versus payment

Hospital guidelines stated that staff should not demand advanced payment for the healthcare that the patient has the right to access. A patient’s ability to pay was an issue to be raised after the treatment had been given, but in advance when the patient had a medical need that was not included within the rights. The OUS guidelines stated that the attending doctor must decide whether the patient has the right to be treated.

The staff at the international office said that the discrepancy between the right to healthcare and the demand for payment was their biggest challenge. In their opinion, the regulation was often interpreted as everyone had the right to have the cost of healthcare covered:

This is where the problem occurs because the right to healthcare is not so big if one, in addition, is to get an invoice for it.

In the guidelines, it was written that the preferred payment method of a self-paying patient was bank card, however, undocumented migrants do not have access to a Norwegian bank card due to their legal status (Figure 2). If payment by bank card was not possible, an address was needed for invoicing. However, undocumented migrants often lack a long-term address. The health secretaries reported that if they could not register an address for the patient, the accounting department had nowhere to send the invoice, and it would just remain unpaid.

### The presumption of an active patient

From the guidelines and the interviews, we found the principle to be that the undocumented migrants were to receive an invoice for the cost of treatment. Several patients reported that they had not been told to go to the counter to pay before they left the outpatient consultation and thus were surprised when they received an invoice afterwards. They felt anxious when learning that they had several outstanding invoices from previous visits. The patients who were unable to pay had to reach out to either the hospital ward or the accounting department, in order to have the invoice credited. In the cover letter instructing self-payment, OUS notified the patient about their right to submit a complaint if they disagreed with the decision: ‘If you believe this decision is incorrect, you have the right to appeal’. However, the patients did not receive any specific information about their rights or about where they could access this information; nor was there a concrete explanation detailing how to submit a complaint. The patients were left to figure this out by themselves.

## Discussion

This is the first exploration of how hospitals navigate between the duty to provide certain forms of healthcare and the financial coverage of treatment to undocumented migrants in Norway. Our study showed that there are practical aspects within the hospitals that are crucial to determining the degree of access to healthcare for undocumented migrants. These aspects include documentation requirements, knowledge and information about the patient’s rights, and how to assess whether a patient should pay for healthcare. Called for by management and administration, this study showed how undocumented migrants are exposed to payment requirements through underlying assumptions, and unclear division of labour and responsibility among hospital staff. Moreover, we found that there is great potential to improve the treatment of undocumented migrants in hospital settings in Norway.

### Othering

The guidelines and everyday practices in the hospital do not in any explicit or systemic way account for the situation of undocumented migrants. In the hospital financial system, this patient group needs to be registered as ‘Other’, that is, as an exception from standardised practices. With significance beyond the registration itself, we suggest that the lack of attention to this patient group in the hospital settings, despite the right to ‘health care that is totally necessary and cannot be deferred’ can be understood as an example of a process of othering. First coined as a theoretical concept by Spivak in 1985 and used in a range of disciplines and academic fields, othering describes discourses, structures and processes that produce and maintain social distinction and distance, in which some are defined as insiders and others as outsiders. As a concept, in the current study, othering included both these discourses, structures and processes, such as the guidelines and practices in the hospitals, and the subject formation involved. Othering produces and maintains both subjects as insiders and in powerful positions and subjects as outsiders and subjugated by these power positions [23]. In migration and postcolonial studies, othering is a way of addressing aspects of race and class in light of European colonialism [15], and points to the ways in which social distinctions are made between people based on power and privilege and how discourses and practices contribute to the marginalisation of minority groups [16]. Othering may also act by condemning poor people for the means they lack or for being poor [24]. Undocumented migrants lack both economic and political power as they are neither consumers nor voters. Hilden and Stålsett have argued that undocumented migrants become the excluded ‘Other’ as they are treated as exceptions in relation to basic human rights in Norway [25]. Their lives have been described as ‘wasted’ by Bauman, who argues that ‘the other’ is an unavoidable side-effect of modernity with economic progress and the quest for order [26].

In this current study, we found that the hospital guidelines did not consider the possibility of foreign patients lacking ID documents, an address or means to pay their bills. ‘Undocumented migrant’ was not a category in hospital register systems and staff had to tick ‘Other’ when registering the patient. Being defined as ‘Other’ meant that there were no adjustments made for that patient, which then required ad hoc navigation from both the patient and the healthcare professionals. It was left up to individual staff to ensure access to healthcare. However, the staff took different positions, such as protecting the hospital expenses or just doing (i.e. invoicing) what was expected of them, or taking the stance that the issue of payment was not their responsibility. All these positions may contribute to producing and maintaining the othering of undocumented migrants within the hospital system. Studies from migration settings have shown how the normality of othering, such as simply filling out the hospital forms available, both creates and classifies some groups to be ‘abnormal’ as well as confirming the hegemonic insider groups [23]. In the case of undocumented patients in hospitals in Norway, othering reproduces the notion of the all-encompassing and generous welfare state.

Several studies have found that health professionals find it challenging to provide proper care to undocumented migrants [10-12]. Reasons for this may be unclear guidelines, feeling restrained by the law or considering ethical dilemmas [13]. Undocumented migrants probably constitute less than 0.5% of the total population in Norway [27], and thus hospital staff have few encounters or learning opportunities to understand how to handle these situations. Undocumented patients may also challenge health professionals’ values and political positions, seen for example in their emphasis on economic issues in their navigation and their wish to protect the Norwegian welfare state, seeing undocumented migrants as undeserving or as a threat [13].

The hospitals’ guidelines not only neglected undocumented migrants but frequently stated that the staff need to see verified documentation about a person’s identity within the record system. The healthcare professionals were also expected to assess whether undocumented migrants’ medical conditions gave them the right to healthcare, an assessment they seldom do with regular patients. Previous studies have found that health professionals interpret and handle the legal text ‘health care that is totally necessary and cannot be deferred’ strictly, and that unclear legislation may lead to postponed or denied care [4]. In a study of undocumented migrants in Norway, Bendixsen has argued that the requirement of documentation and the attached numerous questions to establish the patient’s right to healthcare produces what she calls a micro-practice of border control within the hospital. This, she showed, produces a risk that migrants are refused healthcare [17]. When undocumented migrants are victims of othering within the hospital systems, the hospitals are reproducing the marginalisation experienced in their everyday lives and making their situations more complicated, increasing what can be understood as a structural vulnerability. The concept of structural vulnerability emphasises that adverse health outcomes result from political, social and economic structures rather than individual failure. This includes the various ways healthcare systems and institutions contribute to an individual’s and a population’s vulnerability [28].

The everyday practices of hospital staff are experienced by undocumented migrants as ‘boundary marking – between inside and outside, between deserving and non-deserving and between citizen/legal resident and illegal migrant’, which add to their unbelonging in Norway [17, 29]. To counteract othering requires that hospital staff have the knowledge and capacity to identify and handle structural vulnerabilities. One important way to build this capacity is to address the underlying assumptions of patients’ legal status in the hospital guidelines [30]. Holmes et al. argue that healthcare professionals must ‘critically assess the moral negotiations of deservingness that are inherent parts of their routine clinical and health systems practice to engage more effectively and equitably with migrant patients’ [31], and we hold that this is relevant also for undocumented migrants’ encounters with hospitals in Norway. Sometimes, individual assessments by health professionals may also produce a positive differentiation [4], possibly indicating that healthcare professionals have more room for providing healthcare than they may be aware of.

## Conclusions

The current study found the administrative handling of undocumented migrants to be unsystematic, including the dispersion of responsibility. Both administrative and clinical hospital staff found their skills for assessing undocumented patients’ ability to pay to be very limited or lacking. The underlying assumption of a legally homogenous patient group in the hospitals’ guidelines, with a strict emphasis on personal ID number, documentation and address, contributed to an othering of the undocumented patients and a marginalisation in the health system of individuals who are already living very precarious lives. We recommend that the hospitals reassess their underlying assumptions of patients’ documented status to manage patients without the proper documentation. In addition, we recommend hospital leadership develops greater awareness and capacity among their staff about undocumented migrants’ situations, thus contributing to improving these patients’ access to the healthcare they are legally entitled to.

## Supplemental Material

sj-docx-1-sjp-10.1177_14034948251318512 – Supplemental material for Navigating financial coverage of healthcare to undocumented migrants at two hospitals in Oslo: a qualitative study Supplemental material, sj-docx-1-sjp-10.1177_14034948251318512 for Navigating financial coverage of healthcare to undocumented migrants at two hospitals in Oslo: a qualitative study by Kristine Fjelltorp-Veland, Heidi E. Fjeld and Frode Eick in Scandinavian Journal of Public Health
