# Supplementary material for: Navigating financial coverage of healthcare to undocumented migrants at two hospitals in Oslo: a qualitative study
Source: Scand J Public Health. 2025 Feb 16;54(2):122–30. doi: 10.1177/14034948251318512 (PMC12936140; doi:10.1177/14034948251318512)
Supplement: sj-docx-1-sjp-10.1177_14034948251318512 – Supplemental material for Navigating financial coverage of healthcare to undocumented migrants at two hospitals in Oslo: a qualitative study [file sj-docx-1-sjp-10.1177_14034948251318512.docx]

**Interview guide for hospital staff and volunteers at NGO clinic.**

Background information

- Can you please tell me about your educational background, hospital, and department position?

The encounter with undocumented migrants in your work

- What experiences do you have with undocumented migrants in your work?
- How do you proceed when you meet an undocumented migrant in your work?
  - What questions do you ask?
  - What assessments do you do? (Not medical)
  - How do you relate to the question about payment or their ability to pay?
  - How do you interpret the laws and codes of conduct when treating these patients?
- How does it affect you, or how do you experience the situation when meeting undocumented migrants in your work?
  - What challenges and dilemmas are you experiencing?

Registering undocumented migrants in the hospital systems

- How are undocumented migrants registered in the hospital’s journaling system?
  - Are there several ways to do this? Can you explain the differences?
  - What are your thoughts on the ‘correct’ way to register this patient group?
- What affects if the patient receives a bill for their treatment or not?
- How is the patient’s ability to pay assessed?

Payment

- When does the issue of payment come up?
- Who brings up the issue of payment?
- What information is given to the patient about payment?

For healthcare professionals working at both the hospital and volunteering at NGO clinic

- What differences are you experiencing when meeting undocumented migrants at your work as opposed to meeting them at NGO clinic?

Further questions

- What are your thoughts about undocumented migrants’ access to healthcare in general?
- Do you have any questions, comments, or anything you want to elaborate on?

Ending points for the interview

Thank the interviewee for their participation and contribution. Shortly summarise the information gathered in the interview to make sure that the interviewee agrees. Ask for permission to cite the interview anonymously in the thesis. Remind the interviewee about their rights as an informant in the project and assure them that information is being handled confidentially. Furthermore, remind the interviewee about their right to withdraw from the project at any time and how they can do so.

**Interview guide for undocumented migrants.**

Narrating the course through the hospital

- Can you please tell me about your course through the hospital, starting with your decision to seek help and all the way through to the point where you received a bill and where you went from there?

Questions to guide the participant if needed or to probe more about afterwards:

- What affected your decision to seek medical help?
- What was the urgency for your treatment or consultation?
- Can you tell me about how you were met at the hospital?
  - What questions did you get?
  - How did you experience this meeting?
  - How did the healthcare professionals meet you?
- What information did the hospital give you about payment?
  - When did you first receive information about payment? (Early or late in your treatment course?)
  - Who brought up the issue of payment? And how? (Did it seem random, was it known to begin with or as a part of the hospitals routine?)

Further questions

- How do you experience undocumented migrants’ access to healthcare in general in Norway?
- What are the challenges regarding this access, in your opinion?
- Do you have any questions, comments, or anything you want to elaborate on?

Ending the interview

Thank the interviewee for their participation and contribution. Shortly summarize the information gathered in the interview to make sure that the interviewee agrees. Ask for permission to cite the interview anonymously in the thesis. Remind the interviewee about their rights as a participant in the project and assure the interviewee that information is being handled confidentially. Furthermore, remind the interviewee about their right to withdraw from the project at any time and how they can do so.
